# Tonic signaling of the B‐cell antigen‐specific receptor is a common functional hallmark in chronic lymphocytic leukemia cell phosphoproteomes at early disease stages

**DOI:** 10.1002/1878-0261.70032

**Published:** 2025-03-25

**Authors:** Paula Díez, Pablo Juanes‐Velasco, Marina L. García‐Vaquero, Conrad Droste, Alicia Landeira‐Viñuela, Miguel Alcoceba, Helena Fidalgo‐Gómez, Sara Misiego‐Herrero, Almudena Navarro‐Bailón, Mónica Baile, José M. Bastida, Jose Manuel Sanchez‐Santos, Rafael Góngora, Julia Almeida, Marcos Gonzalez‐Diaz, Alberto Orfao, Javier De Las Rivas, Manuel Fuentes

**Affiliations:** ^1^ Translational and Clinical Research Program, Cancer Research Center (IBMCC, CSIC‐University of Salamanca), Cytometry Service, NUCLEUS, Department of Medicine University of Salamanca (Universidad de Salamanca) Spain; ^2^ Institute of Biomedical Research of Salamanca (IBSAL) Spain; ^3^ Biomedical Research Networking Centre Consortium of Oncology (CIBERONC) Instituto de Salud Carlos III Madrid Spain; ^4^ Proteomics Unit‐IBSAL, Instituto de Investigación Biomédica de Salamanca Universidad de Salamanca (IBSAL/USAL) Spain; ^5^ Bioinformatics and Functional Genomics Group Cancer Research Center (IBMCC, CSIC/USAL), Consejo Superior de Investigaciones Científicas (CSIC) University of Salamanca (USAL), and Instituto de Investigación Biomédica de Salamanca (IBSAL) Spain; ^6^ Department of Hematology, Cancer Research‐Centre IBMCC (CSIC‐USAL, IBSAL) University Hospital of Salamanca, CIBERONC‐CB16/12/00233 Spain; ^7^ Department of Human Anatomy and Embryology, Faculty of Medicine University of Salamanca Spain; ^8^ Department of Statistics University of Salamanca Spain; ^9^ Proteomics Unit Cancer Research Centre (IBMCC/CSIC/USAL/IBSAL) Salamanca Spain; ^10^ Present address: Functional Biology (Immunology area), Faculty of Medicine and Health Sciences University of Oviedo Spain

**Keywords:** B‐cell chronic lymphocytic leukemia, BCR signaling, intracellular protein network, phosphoproteome, proteomics, tyrosine kinase

## Abstract

B‐cell chronic lymphocytic leukemia (B‐CLL) is characterized by highly heterogeneous genomic alterations and altered signaling pathways, with limited studies on its proteome. Our study presents a comprehensive analysis of the proteome and phosphoproteome in B‐CLL and CLL‐like monoclonal B‐cell lymphocytosis (MBL) primary cells. Using high‐resolution mass spectrometry, we identified 2970 proteins and 316 phosphoproteins across five tumor samples, including 55 newly identified phosphopeptides (ProteomeXchange‐PXD005997). Our multifaceted approach also integrated protein microarrays and western blotting for further data validation in a new patient cohort of 14 patients. Despite sharing 73% of their proteomes, the phosphoproteomes varied significantly among samples, independent of cytogenetic alterations and immunoglobulin heavy variable cluster (*IGHV*) mutational status. We identified common functional hallmarks in B‐CLL and MBL phosphoproteomes, notably tonic signaling (low‐level, constitutive signaling) of the B‐cell antigen‐specific receptor (BCR) and nuclear factor NF‐kappa‐B (NF‐kβ)/signal transducer and activator of transcription 3 (STAT3) pathways. Nine phosphoproteins involved in BCR signaling were further validated, showing a high correlation with early disease stages. Our study advances the field by providing a detailed perspective on the proteome and phosphoproteome of B‐CLL cells, revealing signaling pathways crucial for disease development and progression. Integrating diverse proteomics techniques and identifying novel phosphopeptides offers new insights into CLL biology, potentially informing future therapeutic strategies and biomarker development for early diagnosis and personalized treatment.

## Graphical abstract

B‐cell chronic lymphocytic leukemia (B‐CLL) and monoclonal B‐cell lymphocytosis (MBL) show altered proteomes and phosphoproteomes, analyzed using mass spectrometry, protein microarrays, and western blotting. Identifying 2970 proteins and 316 phosphoproteins, including 55 novel phosphopeptides, we reveal BCR and NF‐kβ/STAT3 signaling in disease progression, uncovering new pathways and biomarkers for advancing therapeutic and diagnostic strategies.

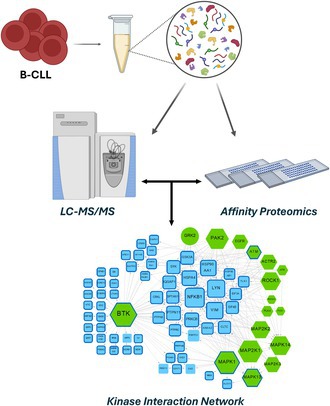

**Abbreviations**
BCR: B‐cell receptor
BSA: bovine serum albumin
CLL: chronic lymphocytic leukemia
DIA‐MS: data‐independent acquisition mass spectrometry
IGHV: immunoglobulin heavy chain variable region
LC–MS/MS: liquid chromatography‐tandem mass spectrometry
MBL: monoclonal B‐cell lymphocytosis
NF‐κB: nuclear factor kappa‐light‐chain‐enhancer of activated B cells
PB: peripheral blood
PTM: post‐translational modification
RT: room temperature
Ser: serine
STAT3: signal transducer and activator of transcription 3
Thr: threonine
Tyr: tyrosine

## 1 Introduction

B‐cell chronic lymphocytic leukemia (B‐CLL) is the most common human blood cancer in the Western world, and it is characterized by high genomic heterogeneity in the absence of a common genetic lesion [1, 2]. In recent years, both the genome of the CLL B cells [3, 4, 5, 6] and the proteome [7, 8, 9] have been investigated in great detail, although its impact on signaling pathways is not fully understood. As in most human cancers, the understanding of malignant transformation cells is guided by dysregulation/alteration of intracellular protein networks, including protein kinase signaling pathways [10, 11]. In turn, in CLL such altered protein networks might be targets for small molecules as innovative therapies [7, 9, 12, 13].

Likewise, protein phosphorylation is a key post‐translational modification regulating protein function in many cellular processes [14, 15]. In that sense, it is known that the genetic alterations and DNA damage associated with CLL trigger changes in the phosphorylation patterns of specific substrates [7]. These, in turn, modify protein stability, localization, protein–protein interactions, and enzymatic activity, which ultimately have a direct impact on B‐cell signaling [12, 16, 17]. Currently, it is well established that the tonic B‐cell receptor (BCR) signaling pathway plays a critical role in the development of CLL, leading to altered phosphorylation patterns of specific proteins related to cell proliferation, differentiation, and survival [9, 18, 19, 20, 21]. Moreover, it is central in the crosstalk with other functional pathways, such as in regulating cytokine signaling, microtubule dynamics, or cell–cell interactions. These functions are coordinated by signal transduction cascades that involve adapters, second messengers, and tyrosine kinases, such as LYN and SYK [22, 23]. LYN phosphorylates activation motifs at multiple and different phosphosites on the alpha and beta chains of the immunoglobulin molecules, whereas the SYK tyrosine kinase, together with several phosphatases, generates an inhibitory effect on the signal transduction of the BCR pathway [24].

Due to its central role in B‐cell physiology, the inhibition of BCR signaling pathways has become the leading treatment strategy across a variety of lymphoid malignancies [3]. In recent years, the therapeutic strategies for CLL have undergone great improvement thanks to the development of new small molecules for the inhibition of BTK and BCL‐2 proteins, such as ibrutinib, acalabrutinib, or venetoclax [25, 26]. However, there are still open issues regarding the indirect impact of these drugs on other cells from the microenvironment and the mechanisms of resistance of CLL cells. On the contrary, although very promising, anti‐CD19 CART cell‐based immunotherapies are still in the early stages of implementation and still require refining dose–response ratios to balance their efficiency and toxicity (i.e., lymphodepletion regimen and infused cell dose) [27]. In addition to advanced treatments with minimum side effects, clinicians require biomarkers for early CLL diagnosis and to apply effective therapeutic algorithms according to the molecular profile of each patient. Most human cancers are triggered by the dysregulation of protein networks, including protein kinase signaling pathways [7, 8, 9, 10, 11]. Thus, the simultaneous characterization of CLL proteome and phosphoproteome would help to identify the signaling pathways distinctively involved in the pathological expansion and survival of B cells [12, 13, 16].

In that sense, previous studies have proved that the combination of proteomics approaches for characterizing CLL cells with different cytogenetic alterations is useful in deciphering CLL biology [28, 29, 30]. Similarly, global quantitative proteome changes have been reported by Alsagaby et al. [31] by applying iTRAQ technology in 12 CLL samples, demonstrating the presence of alterations in spliceosome and proteasome pathways (i.e., EPB4.1, CNN3, H1‐4, HIST1H4G, Rab8b, Ikzf3, and GNA13), compared with independent normal B cells. Recently, the proteome of CLL cells was analyzed by data‐independent acquisition mass spectrometry (DIA‐MS) reporting a protein landscape of 3314 proteins that identified limited protein abundance buffering and an upregulated protein complex involved in BCR, AKT, MAPK, and PI3K signaling in trisomy 12 CLL [9]. Overall, these studies show the usefulness of proteomic approaches, especially when combined with transcriptomic data to specifically identify disease‐related biomarkers. In the last years, it has been reported that several phosphoproteomics profiling of CLL patients as well as systematic kinases studies are opening a new hallmark in understanding CLL pathology and disease evolution [32, 33, 34, 35, 36, 37, 38].

The characterization of the phosphoproteome profile of a given cell/cell population may be considered a challenge, mainly due to the complexity of the distinct pathways involved, the low levels of stoichiometry, and the high dynamic range of phosphopeptides [16, 39]. In this regard, phosphosite‐enrichment strategies are highly valuable, particularly those based on specific antibodies against targeted phosphosites/phosphopeptides [40]. For instance, mass spectrometry (MS)‐centric phosphoproteomics has been consolidated as a key tool for the profiling of global post‐translational modifications (PTM) due to its high sensitivity and the ability to simultaneously detect thousands of single amino acid modifications per assay [39]. Moreover, the refinement of label‐free MS strategies—that is without isotopic labeling—allows quantitative comparisons between the relative amounts of the targeted phosphoproteins/phosphopeptides, avoiding additional costs and tedious experimental procedures [41]. Thus, we propose using multifaceted proteomic approaches to generate detailed prospects in the physiology of CLL cells. The investigation of the CLL functional profile can be used to propose pathological intracellular pathways for further verification. The validation of protein candidates can be performed by affinity proteomics, which allows the simultaneous specific detection of many proteins in a single assay employing a minimal amount of sample [28].

In the present study, the proteomes and phosphoproteomes of 13qCLL and monoclonal B‐cell lymphocytosis (MBL) primary tumor B cells using high‐resolution MS‐based approaches have been investigated. Overall, we identified 13 504 peptides from a total of 2970 quantified proteins and 594 phosphopeptides from 316 phosphoproteins, of which 55 corresponded to novel phosphopeptides reported here for the first time. Most of these phosphoproteins were involved in alterations of the BCR signaling, as well as some routes of B lymphocyte activation, which are highly correlated with the CLL therapeutic algorithm and disease progression. Here, nine of these proteins have been verified by affinity proteomics and conventional immunoblotting, showing relationships between these phosphoproteins and early stages of the disease and *IGHV* mutational status, which provide new insights into the global proteome and phosphoproteome of CLL and the immunological signaling pathways involved in tumor development and progression at the tumor cell level. Finally, our phosphoproteomic analysis revealed promising avenues for drug repurposing in CLL treatment, which might be the basis for new promising therapeutic approaches.

## 2 Materials and methods

### 2.1 Patients and samples

Isolated B cells from peripheral blood (PB) samples by FACS sorting (as described by Diez et al. [42]) were obtained from 19 (18 CLL patients and 1 CLL‐like MBL) patients (Table 1 and Table S1) at University Hospital of Salamanca (June 2016 to April 2019). The experiments were undertaken with the understanding and written consent of each subject according to the guidelines of the local ethics committees of the University Hospital of Salamanca (code reference: CEIm PI 2021 02694), the National DNA Bank‐ISCIII, and the Declaration of Helsinki of 1975, as revised in 2008. In each case, fresh PB‐derived tumor (CD19^+^CD5^+^) B cells were purified (purity > 98%) using a FACSAria III (BD, San Jose, CA, USA) flow cytometer at the Cytometry Service of the University of Salamanca (NUCLEUS, Salamanca, Spain) [43]; FACS‐sorted tumor B cells were immediately stored frozen in liquid nitrogen at the National DNA Bank‐ISCIII (Salamanca, Spain), until analyzed as described below.

**Table 1:** Patient's samples description. Clinical features of the 19 CLL/MBL samples analyzed in this study, including patient age, sex (M: male; F: female), diagnosis, % of clonal population from WBC (WBC: white blood cells), Binet/Rai stage, V(D)J rearrangement, *IGHV* mutational status (*IGHV*: immunoglobulin heavy variable; UM: unmutated; M: mutated), cytogenetic alterations and CD38 and ZAP70 status. N.A., information not available.

Sample ID	Age	Sex	Diagnosis	%clonal population (from WBC)	Binet stage	Rai stage	V(D)J rearrangement	*IGHV* mutational status	Cytogenetics	CD38	ZAP70
A	53	M	CLL	90%	B	I	V1‐3/D6‐19/J4	UM	del13q14 (D13S25)	0	N.A.
B	71	M	CLL	79%	B	II	V1‐2/D3‐9/J6	UM	del13q14 (D13S25), tr12, del14q32	100	100
C	72	F	CLL	90%	C	IV	V3‐9/D3‐10/J6	UM	ATM 97%, del13q 93%, del14q32 14%	30	24
D	81	M	CLL	92%	C	IV	V1‐18/D2‐2/J3	M	del13q14 (D13S25 + RB1)	0	28
E	76	F	CLL‐like MBL	28%	B	I	V4‐4/D6‐19/J4	M	RB1	50	N.A.
F	92	M	CLL	77%	A	0	V5‐51/D2‐2/J6	M	del13q14 (D13S25) (97%)	N.A.	N.A.
H	74	F	CLL	91%	A	II	V3‐23/?/J6	UM	del13q14 (D13S25 (99%) + RB1 (99%))	0/10	0/0
J	57	F	CLL	60%	A	0	V4‐34/D4‐23/J6	M	del13q14 (D13S25) (99%)	0	0
K	83	F	CLL	86%	B	III	V1‐18/6‐19/4‐02	UM	del13q14 (D13S25)	50	0
L	81	M	CLL	77%	A	0	V6‐1/D6‐19/J4	M	del13q14 (D13S25)	0	0
M	61	M	CLL	69%	A	0	V3‐23/D2‐15/J4	M	del13q14 (D13S25)	0	0
N	55	F	CLL	95%	A	0	V1‐8/D3‐3/J6	UM	del13q14 (D13S25 + RB1)	59	59
O	75	F	CLL	91%	B	III	V3‐30/?/J6	UM	del13q14 (D13S25 (99%) + RB1 (99%))	0/10	0/0
P	81	M	CLL	72%	A	0	V3‐30/D3‐3/J6	UM	del13q14 (D13S25 + RB1)	75	8
Q	71	M	CLL	76%	A	0	V1‐69/2‐02/6‐02	M	del13q14 (D13S25)	0	0
R	66	M	CLL	94%	C	III	V3‐23/D3‐9/J6	UM	del13q14 (D13S25 + RB1)	0	22
S	N.A.	M	CLL	79%	N.A.	N.A.	V4‐31/D3‐3/J6	UM	del13q14 (D13S25)	N.A.	N.A.
T	86	M	CLL	87%	A	0	V3‐23/D2‐2/J4	M	del13q14 (D13S25 + RB1), tr12, del14q32	90	51
V	65	F	CLL	72%	B	II	V1‐69/D3‐3/J6	UM	del13q14 (D13S25 + RB1), tr12, del14q32	53	83

### 2.2 Sample preparation

Depending on the different proteomics characterization strategies to be applied, each sample was processed according to the optimal protocol for each proteomics assay described below:

#### 2.2.1 Characterization by LC–MS/MS

Freshly frozen B cells were thawed, pelleted by centrifugation at 200 ***g*** (5 min at 4 °C) and washed three times with PBS. After draining off the total PBS volume without disturbing the cell pellet, 1 mL (per 1.25 × 10^8^ cells) of a urea‐based lysis buffer – 9 m urea, 1 mm activated sodium orthovanadate, 2.5 mm sodium pyrophosphate (both acting as phosphatase inhibitors), 1 mm β‐glycerol phosphate, 20 mm HEPES pH 8.0; all from Sigma, St. Louis, MO, USA – was added to the cell pellet, followed by sonication on ice (3 bursts for 30 s each). Then, samples were centrifuged at 20 000 ***g*** for 15 min and the supernatant containing the proteins was collected [2] and stored at −80 °C until used. Protein concentration was determined by the Bradford assay using the Coomassie Plus Protein Assay Reagent (Thermo, Waltham, MA, USA).

#### 2.2.2 Characterization by affinity proteomics

Freshly frozen B cells were thawed, pelleted by centrifugation at 150 ***g*** (5 min at 4 °C) and washed with PBS. After draining off the total PBS volume without disturbing the cell pellet, 500 μL (per 10 × 10^6^ cells) of lysis buffer—5 m NaCl, 0.5 m EDTA, 100% glycerol, 100% NP40, 1 m Tris‐HCl pH 7.0, and 2% protease/phosphatase inhibitors (Halt™ Protease and Phosphatase Inhibitor Cocktail); all from Thermo Fisher Scientific™—were added to the cell pellet, followed by 15 min of incubation at 4 °C. Then, samples were centrifuged at 15 800 ***g*** for 15 min at 4 °C, and the supernatant containing the proteins was collected and stored at −80 °C until used. Protein concentration was determined by the Pierce™ BCA Protein Assay Kit (Thermo Fisher Scientific™). Before incubation on microarrays, proteins were biotinylated (0.7 μL NHS‐PEG4‐biotin per 6 μg protein) following the protocols described by Haggmark et al. [44] and Henjes et al. [45]. Briefly, proteins were incubated for 2 h on ice, and then, the reaction was stopped with 0.5 m Tris–HCl pH 8.0 for 20 min at room temperature (RT) and stored at −20 °C until use.

### 2.3 Phosphorylation characterization by LC–MS/MS following the PTMScan method

**Fig. 1:**
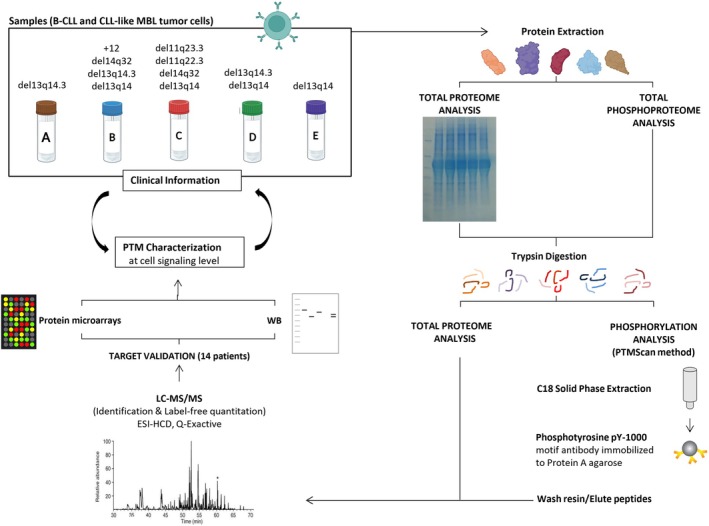
General workflow used in this study. From global proteomics and phosphoproteomics analyses to validation through affinity proteomics (protein microarrays) and WB. B‐CLL, B‐cell chronic lymphocytic leukemia; CLL, chronic lymphocytic leukemia; ESI‐HCD, Electrospray Ionization (ESI) with Higher‐energy Collisional Dissociation; LC–MS/MS, liquid chromatography tandem mass spectrometry; MBL, monoclonal B‐cell lymphocytosis; PTM, post‐translational modification; WB, western blot.

After protein extraction, a phosphorylation characterization by LC–MS/MS was performed (Q‐Exactive HF; Thermo) following the steps of the PTMScan method. Each protein sample (15 μg) was separated on a 4–20% gradient SDS/PAGE gel under reducing conditions. After electrophoresis, gels were stained in a Coomassie solution (Fig. S1). Then, each gel line was cut into gel pieces that were destained with 15 mm potassium ferrocyanide and 50 mm sodium thiosulfate (both from Sigma). Proteins (in solution and from gel pieces for phosphorylation and total proteome assays, respectively) were reduced in 4.5 mm DTT (Sigma) for 30 min at 45 °C and alkylated with 100 mm iodoacetamide (Sigma) for 15 min at RT in the darkness. Afterwards, protein digestion was performed overnight in 10 μg·mL^−1^ trypsin (Promega, Madison, WI, USA). The peptide solution was acidified with 1% trifluoroacetic acid (TFA, Sigma) and desalted with 360‐mg SEP‐PAK Classic C18 columns (Waters Corp., Milford, MA, USA). Peptide elution was performed with 40% acetonitrile (Sigma) in 0.1% TFA, and peptides were dried under vacuum conditions. Lyophilized peptides were mixed with PTMScan® Direct reagent bead slurries (phosphotyrosine pY‐1000 motif antibody; Cell Signaling Technology, Danvers, MA, USA) for 2.5 h at 4 °C [31]. Afterward, beads were pelleted and washed, and the peptides were eluted from the beads with 0.15% TFA. Enriched peptides were purified on C18‐Stage‐Tips columns (Thermo) and stored at −20 °C until analyzed by LC–MS/MS. Figure 1 and Fig. S1 display a schematic illustration of the workflow of the whole procedure.

### 2.4 Characterization by affinity proteomics and western blot

A total of 208 antibodies (100 μg·mL^−1^ per antibody) targeting 162 proteins (Table S2) were deposited in triplicate onto an amino‐silanized surface by using a noncontact printer (ArrayJet®Printer Marathon v.1.4), according to the protocol described by Gonzalez‐Gonzalez et al. [46] (Fig. S2A). Antibody corresponding solutions (MasterMix‐MM), and positive and negative controls, were also printed in triplicate (Table S2). The sample incubation on the protein microarrays was performed as follows: 100 μL of each biotinylated B‐CLL cell lysate (at 0.1 μg·μL^−1^) was added to the microarrays and incubated overnight in agitation following the protocol described by Haggmark et al. [44]. Then, the microarrays were washed 3× for 5 min with 0.5% PBST (phosphate‐buffered saline, 0.1% Tween 20) followed by the addition of 2 μg·mL^−1^ streptavidin‐Cy5 (200 μL per array, 30 min at RT in the darkness). After three washing steps, the microarrays were dried out by centrifugation (3 min, 110 ***g***) [47, 48].

Array images were acquired by SensoSpot Fluorescence scanner (Sensovation AG, Miltengyi Imaging, Berlin, Germany) and analyzed using genepix®pro 6.0 software (Fig. S2B). First, data processing, filtering, and preprocessing (background subtraction) were carried out to eliminate variations due to printing, fluorescence, and image acquisition so that only real biological variation is preserved. Finally, data were normalized according to the trimean of the positive controls [49].

For western blot, 25 μg of each CLL cell lysate sample were resolved on 12% gradient SDS/PAGE gels under reducing conditions. Then, proteins were transferred to activated PVDF membranes (Immobilon‐P PVDF, Sigma‐Aldrich) at 400 mA for approximately 2 h. Afterwards, membranes were blocked for 1 h in 5% (w/v) BSA blocking solution (BSA and 0.1% PBST) followed by overnight incubation at 4 °C and mild stirring with the corresponding primary antibody at a 1 : 900 (v/v) dilution [i.e., 13 monoclonal rabbit antibodies targeting human tyrosine phosphoproteins (Table S2)]. Then, the membranes were washed 3× for 7 min with 0.1% PBST. After that, the rabbit anti‐IgG HRP‐conjugated secondary antibody at a 1 : 5000 (v/v) dilution was added for 1 h incubation. After three washing steps, membranes were incubated with SuperSignal West Pico Luminol/Enhancer Solution and SuperSignal West Pico Stable Peroxide Solution (Thermo Fisher Scientific™) for 1 min and revealed in the dark chamber with Fuji X‐ray films Super RX‐N (Fujifilm, Tokyo, Japan).

### 2.5 Integration of quantitative proteome characterization with transcriptome data set

The quantitative proteome profiles from LC–MS/MS experiments of CLL samples A, B, C, and D were cross‐validated with microarray expression profiles from CLL tumors and B‐cell normal samples available at Gene Expression Omnibus database (GEO accession GSE50006) [50], as it is described in Fig. S3. The 188 CLL and 32 B‐cell normal samples were normalized as suggested by authors using RMA and COMBAT algorithms (available at affy and sva R packages, respectively) [51, 52]. To improve protein mapping, the 19 939 genes—in ENSG ID format—included in the microarray platform and the 2970 proteins detected by LC–MS/MS—as Uniprot IDs—were mapped to 18 028 and 2922 Symbol IDs, respectively, using biomart r package [53]. From the 2922 Symbol genes identified in at least one LC–MS/MS sample, 89.3% were also detected in the microarray profile. From these, 82.83% of the validated protein‐coding genes were detected across all four LC–MS/MS experiments. Both rocket plot and density plot are shown in Fig. S3, revealing the proteins detected across A, B, C, and D samples which tend to be expressed at higher levels in the microarray profile.

### 2.6 Kinase–substrate interaction network retrieved from NetworKIN database

Kinase signaling network was retrieved from NetworkKIN database (September 2020) [54]. NetworKIN incorporates both experimentally validated and motif‐based predicted kinase–substrate interactions [54, 55]. The network only includes the interactions between kinases and substrates detected in all CLL samples from the quantitative proteome and phosphoproteome profiles.

## 3 Results

### 3.1 Proteome analysis of 13qCLL and MBL cells

#### 3.1.1 Quantitative proteomic profile of 13qCLL cells

The protein content of 13qCLL cells was determined for each of the five samples isolated from patients, running two technical replicates for each sample within the LC–MS/MS proteomic platform, as described in Materials and methods (Table S3). A total of 13 504 unique peptides were identified corresponding to 2970 unique proteins. Of these proteins, 28 corresponded to proteins with no evidence at the protein level in the current version of the human proteome: 25 have evidence at the transcript level (PE = 2), 1 was inferred by homology (PE = 3), 1 was a predicted protein (PE = 4), and 1 was an uncertain protein; according to the neXtProt database (January 2020 release) and following the HUPO guidelines (www.hupo.org).

**Fig. 2:**
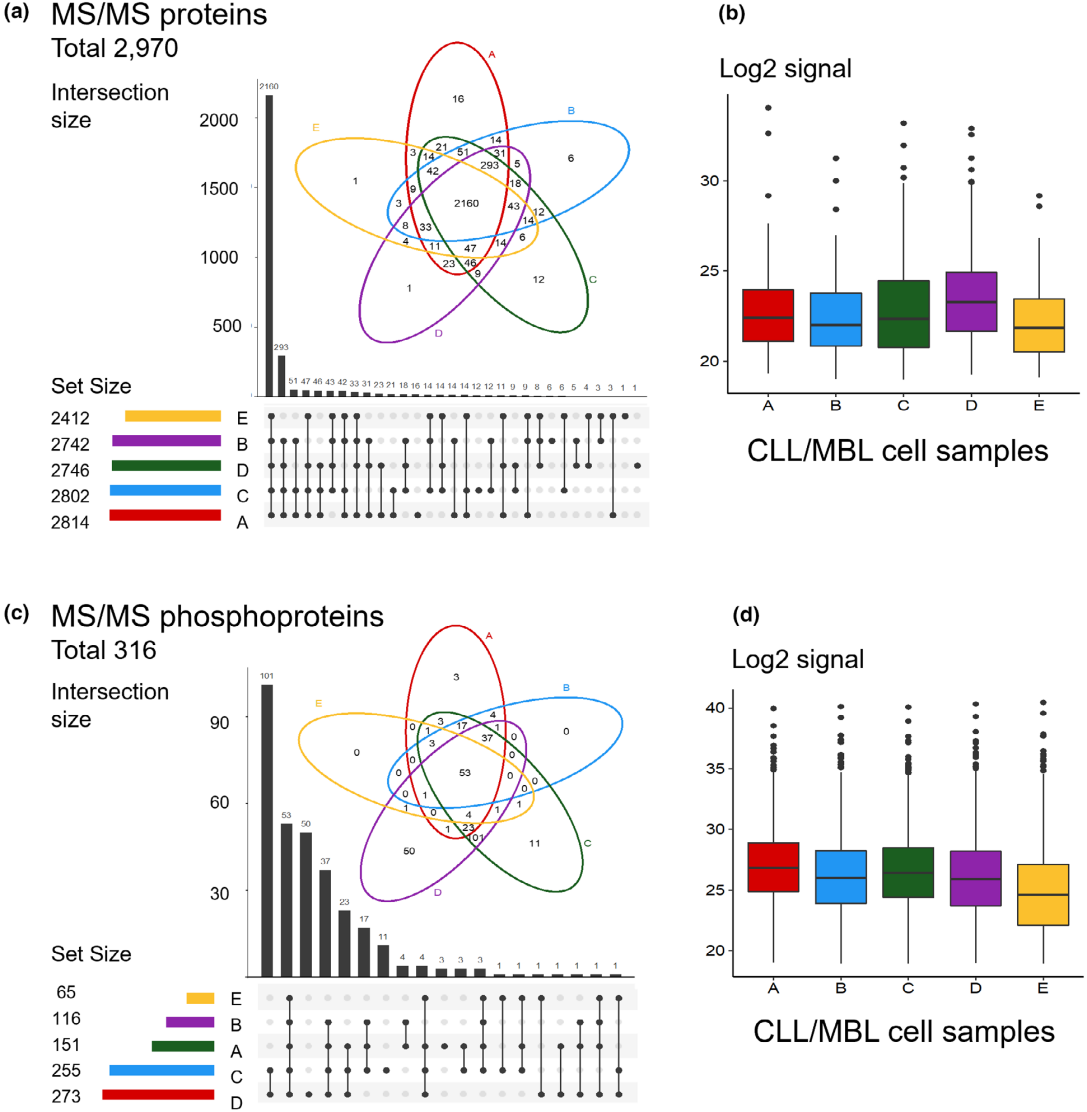
Proteins detected in the proteome and phosphoproteome of CLL and MBL samples. (a, c) Venn diagrams and UpSet plots summarizing the common proteins and phosphoproteins identified by LC–MS/MS along four CLL samples (A, B, C, D) and one MBL sample (E). (b, d) Box plots describing LC–MS/MS log2 signal distribution of proteins and phosphoproteins detected in four CLL samples (A, B, C, D) and one MBL sample (E). Error bars indicate standard error of the mean. CLL, chronic lymphocytic leukemia; MBL, monoclonal B‐cell lymphocytosis; MS/MS, tandem mass spectrometry.

Once we compared the proteins identified per sample, a high degree of correlation and similarity was observed both in the comparison of the five pairs of replicates (i.e., samples A1 vs. A2, B1 vs. B2, C1 vs. C2, D1 vs. D2, and E1 vs. E2, which provided correlations ≥ 0.98) (Fig. S4A), and also in the pairwise comparison between samples from different patients (40 comparisons with correlation values ≥ 0.93). The global distribution of proteins (Fig. 2a) showed a high consistency across the B‐cell samples, with 73% of the total 2970 proteins found in common. Furthermore, the LC–MS/MS quantification of the proteome of the 13qCLL patients revealed similar log2 signal distributions in all samples, with the exemption of sample D (Fig. 2b), which corresponds more properly to a high‐count monoclonal B‐cell lymphocytosis (MBL), which is clinically similar to a very early stage of CLL [56].

The functional enrichment analysis (Table S4) of the common 2160 proteins expressed in 13qCLL/MBL tumor cells returned 154 functional hallmarks. As expected, many of the functional clusters were associated with general cellular functions and previously reported pathways for CLL (e.g., metabolic and catabolic processes, cell cycle, cell–cell adhesion, and RNA transport, among others). Moreover, several clusters were associated with specific B‐cell functional roles in the immune response, such as antigen processing and presentation, functions related to the complement system and the MHC class II molecules, and BCR signaling.

#### 3.1.2 13qCLL/MBL tumor cell protein profiles and other 13qCLL/MBL cellular features

**Fig. 3:**
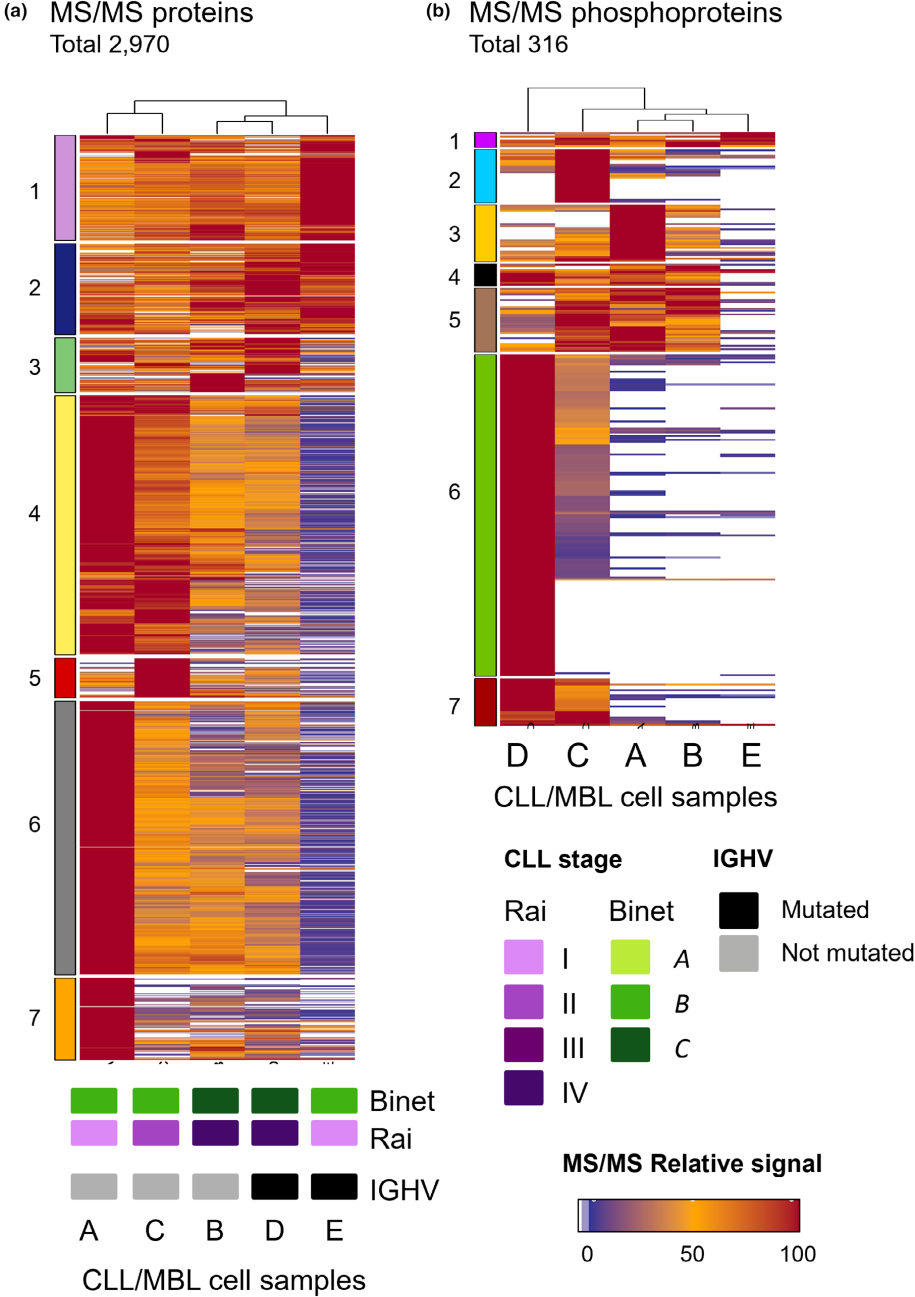
Quantitative protein expression profiles of the proteome and phosphoproteome of CLL and MBL samples. (a, b) Heatmaps representing the relative expression signal of the proteins quantified in the LC–MS/MS experiments for the proteomes and phosphoproteomes identified in four CLL samples (A, B, C, D) and one MBL sample (E). The column dendrograms show sample similarities and the vertical colored‐boxes indicate the protein groups retrieved by hierarchical clustering. An expression signal color scale is included in the figure, corresponding white to the not expressed, and from purple to dark red increasing expression. CLL, chronic lymphocytic leukemia; *IGHV*, immunoglobulin heavy chain variable region; MBL, monoclonal B‐cell lymphocytosis; MS/MS, tandem mass spectrometry.

As stated above, based on the overall proteome of 13qCLL/MBL cells, two main groups of samples were observed (Fig. 3a): Group 1 included samples A and C, and Group 2 consisted of samples B, D, and E (although E, as earlier mentioned, is also quite different from B and D). These groups were identified by the hierarchical cluster analysis (performed with the hclust algorithm), which measures the similarity of the protein expression profiles across the five samples (i.e., the quantitative signal of the 2970 proteins included in our proteomic study). Analysis of these profiles also provided a clustering of the proteins into seven groups (indicated in Fig. 3a) that included the proteins with the most similar profiles. In Table S5, we include the names of the specific proteins present in each cluster in the same order as they are represented in the heatmap. Functional enrichment analysis of the clusters in these heatmaps reveals a clear signature that marks leukocyte alterations and neutrophil dysregulation for the largest cluster (Fig. 3a, Cluster 6). More specifically, this cluster presents a strong alteration of the proteasome activity (with over‐expression of PSMA1, PSMA2, PSMA4‐6, PSMD1, PSMD3, PSMD6‐9, and PSMD11‐14, observed in sample A but also C, B and D). The clustering also shows a clear modulation of many lymphocyte‐associated proteins, for example, related to HLA processing, antigen presentation, and immune regulation (e.g., BCLF1, IgHM, CD2‐P, CD5, CD47, CD48, CD53, CD79B, IGBP1, IGHC, IGHG1, B2M, and ZAP70), HLA proteins (‐A, ‐C, ‐DMB, ‐DRB1, and ‐E), and the BCR signaling pathway (SYK, LSP1, MYCBP, BLNK, and ATM).

**Fig. 4:**
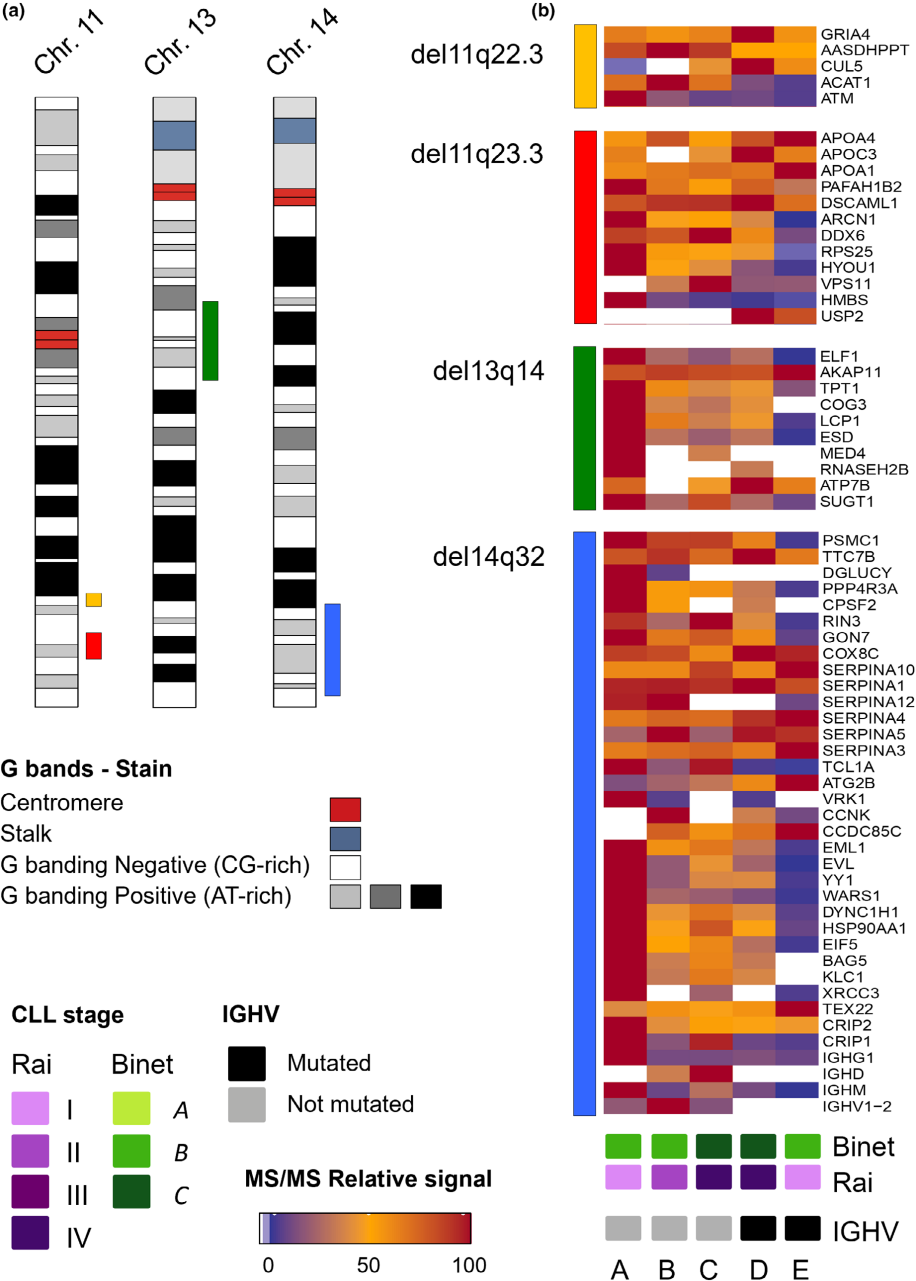
Quantitative LC–MS/MS profiles of the proteins involved in the most relevant cytogenetic regions for CLL prognosis located in chromosomes 11, 13, and 14. (a) Illustrative representation of chromosomes 11, 13, and 14. Vertical colored bars indicate the approximate regions depicted in the heatmaps included in Panel (b). (b) Heatmaps representing the relative signal quantified at LC–MS/MS experiments for the proteins coded by genes localized at four chromosomal regions (i.e., del11q22.3, del11q23.3, del13q14, and del14q32) that are most relevant for CLL prognosis. Bottom colored boxes indicate patient CLL stage (as provided by the Rai and the Binet categories) and *IGHV* mutational status (Mutated or Not Mutated). CLL, chronic lymphocytic leukemia; *IGHV*, immunoglobulin heavy chain variable region; MS/MS, tandem mass spectrometry.

In contrast, it was not directly observed a clear association between the cell cytogenetic alterations, *IGHV* mutational status, and the overall tumor cell protein expression profiles (Fig. 4). Notwithstanding, we found evidence for dysregulation of expression of proteins coded in the chromosomal regions frequently associated with CLL progression, namely del11q22.3, del11q23.3, del13q14, and del14q32 (Tables S6 and S7). For instance, lower expression levels of CUL5 were associated with del11q22.3 (sample B), whereas lower expression levels of ATM were also associated with del11q22.3 (Cases B, C, D and E). In addition, decreased amounts of ARCN1, RPS25, and HMBS were found in sample E that carried del11q23.3 (Fig. 4). Similarly, the expression levels of CD20, CD5, CD45, CD23, CD79B, CD49D, and CD200 identified by MS–MS correlated with the immunophenotypic profile found at the single‐cell level by flow cytometry (as previously described by Diez et al. [30]).

### 3.2 Phosphoproteome analysis of 13qCLL cells

#### 3.2.1 Tyrosine‐phosphoproteome profile across distinct 13qCLL tumor B‐cell samples

The phosphoproteome profile of 13qCLL/MBL tumor cells was evaluated after applying an affinity enrichment method for selective and specific detection of phosphotyrosine motifs (P‐Tyr‐1000; PTM) present in the overall protein fraction of each CLL or MBL cell sample. In addition to this PTM selection, phosphorylation of threonine (Thr) and serine (Ser) was also identified but in minor numbers compared with P‐Tyr due to the specific and selective immunoenrichment performed in this study.

Overall, 594 phosphopeptides corresponding to a total of 316 phosphoproteins were identified across the A, B, C, D, and E samples (Fig. 2c and Table S7), whereas from the 2160 proteins commonly expressed in the tumor B cells, 220 (10%) were found phosphorylated (Table S3). Between these proteins, BTK, PRKCB, STAT1, and SYK might be highlighted as they are key players in the BCR pathway. Additionally, proteins involved in the regulation of the cell cycle (e.g., ATM and STAT3), cell proliferation (e.g., LYN and SRRT), metabolism (e.g., GOT2, PPP1CA, and PYGB), and the regulation of Ca2+ flux (e.g., ANXA6 and CALM1) were also found phosphorylated in the tumor cells. On the contrary, 20 functional clusters were related to signaling pathways associated with B and T cells and to the proteasome (e.g., PSME3, PRKACA, UCHL5, and PSMD14) (Table S4). In contrast, phosphorylated proteins whose expression was restricted to a single sample were limited to a small number of functional pathways, including DYNLL1 (involved in apoptosis) and NBN (involved in cellular response to DNA damage) for sample A; RFWD3 (protein ubiquitination) for sample B; PIK3R4 (autophagy of peroxisome) and ATRN (involved in chemotactic activity of chemokines) for sample C; and ARIH2 (involved in the pathway protein ubiquitination) for sample E. Also, as can be seen in Fig. 2d, the phosphoproteome of sample E that corresponds to MBL provided a much lower signal, and only 65 phosphoproteins were detected (Fig. 2c). Of note, 55 of the total 594 phosphopeptides identified in these five samples were identified for the first time, and therefore, they had not been previously reported in the neXtProt database (January 2020 release). Likewise, 69 of the total 316 (21.8%) phosphorylated proteins were only identified via the enrichment method since they had not been detected by the quantitative global proteome analysis (Table S7). These phosphoproteins included two proteins with only experimental evidence at the transcriptional level (PE = 2) in the neXtProt database, which were the olfactory receptor 4K13 and the uncharacterized protein C11orf53.

The global comparison of the samples A–E showed a high correlation between replicates (*r* ^2^ > 0.99, Fig. S4); but a lower correlation between different samples (*r* ^2^ between 0.63 and 1.00). In the same way, 53 out of the 316 phosphoproteins (17%) (Fig. 2c) were detected in common in the 13qCLL/MBL cells. These phosphoproteins (Table S7) corresponded to proteins directly involved in BCR signaling (protein kinases, such as PRKCB, LYN, SYK, ATM, BLK, JAK1, and JAK2), signal transduction (e.g., KHDRBS1, STAM2, and STAP1), and intracellular protein transport (NSF and CUL3) (Table S4). The analysis also revealed that most of the phosphoproteins were present only in a subset of the samples or just in one sample. For example, 101 out of the 316 phosphoproteins (32%) were uniquely phosphorylated in samples C and D (e.g., BLK, CD19, PLCG2, and SCIMP). The functional analysis of this subset showed that these proteins had specific roles in platelet activation, RNA binding, and spliceosome. Moreover, they contained relevant interaction domains, such as the SH3, SAP, KH, and LIM domains (Table S4). In turn, sample A showed three uniquely phosphorylated proteins (HERC1, NUDT3, and PSMD9) and 11 and 50 proteins were restricted to sample C (DCP1B, GGA2, GBE1, IFIT5, JAK1, PPA2, SRRT, SRSF7, XRCC6, and ZNF24) and sample D (CBL, DOCK11, DOK2, EXOSC10, IKZF3, ILF3, LSP1, PDCL3, PTPN18, RIPK2, SASH3, SETD1A, and YBX1), respectively (Table S7). Finally, the phosphoproteome clustering (Fig. 3b) revealed a great involvement of these cells (Cluster 6, especially for sample D) in the regulation of mRNA splicing via spliceosome (with enrichment in GO terms: GO:0048025, GO:0033119, GO:0050686, and GO:0045292), all related to splicing and RNA processing. Splicing disorders and aberrant splicing have been reported to be characteristic features of CLL cells [57].

As described above, the proteomic profiles of the 13qCLL samples (A–D) showed a significant overlap (Figs 2a,b and 3a) but the phosphoproteome profiles revealed notably higher heterogeneity (Figs 2c,d and 3b). Overall, the phosphoproteomic signature indicated the presence of two groups of samples (Group 1: samples A B and E; Group 2: samples C and D; Fig. 3b), characterized by the differential expression profiles of seven main groups of proteins (Fig. 3a and Table S5). The contributing group of proteins consisted of 175 proteins (Cluster 6). These included the phosphorylated LYN, SYK, BLK, LSP1, RAB10, and BTK, among other proteins. The remaining clusters (1, 2, 3, 4, 5 and 7) contributed less to the clustering (Fig. 3a,b). No clear association was found between the tumor cell phosphoproteome and tumor cytogenetics, the *IGHV* mutational status, and other clinical features.

### 3.3 Validation of phosphoprotein profiles in BCR signaling and across 13qCLL disease stages by affinity proteomics and western blot

**Fig. 5:**
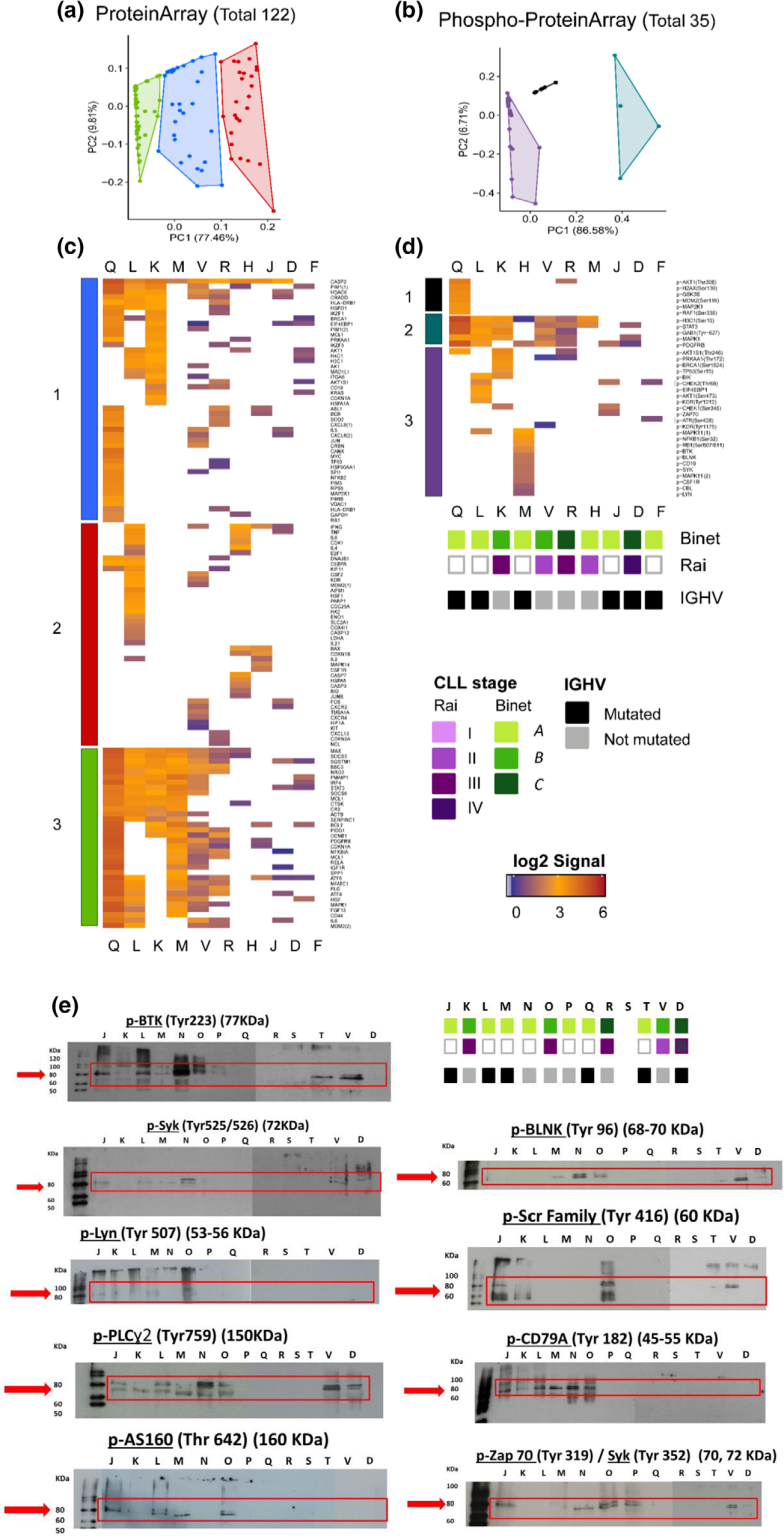
Proteome and phosphoproteome profiles of CLL samples using protein microarrays and western blot validation. (a, b) Principal component analysis of the proteins and phosphoproteins detected with the protein microarrays in the new set of 10 CLL samples. (c, d) Heatmaps representing the log2 signal of the proteins (total 122) and the phosphoproteins (total 35) detected with the microarrays. Vertical colored bars (1–3) indicate the sample groups identified at the multidimensional analyses on Panels a and b. Bottom colored boxes on Panel d indicate the patient CLL disease stage (as provided by the Rai and the Binet categories) and the *IGHV* mutational status (mutated or not mutated). Samples at both heatmaps are arranged in the same order; thus, the clinical information about the disease category is indicated below the heatmap in Panel d (and it is applicable to Panel c). (e). Western blot results from nine phosphoproteins involved in BCR signaling in 13 CLL cell lysate samples. Twenty‐five micrograms were resolved on 12% gradient SDS/PAGE gels under reducing conditions. Red arrows indicate the predicted molecular weight for each protein. Letters refer to patient samples as defined in Table 1. CLL, chronic lymphocytic leukemia; *IGHV*, immunoglobulin heavy chain variable region.

To confirm these findings in a larger cohort of patients, an independent set of 10 13qCLL samples (Table S1) was analyzed by a customized protein microarray containing 208 antibodies targeting 157 proteins and phosphoproteins previously observed in the quantitative proteome landscape (Table S2). Then, protein profiles of these 13qCLL samples were compared side‐by‐side to distinguish the common and specific hallmarks of each CLL sample (Fig. 4). From the 122 proteins identified in the protein microarray, we corroborated the expression of several proteins involved in the BCR signaling pathway (LYN, SYK, BTK, and ZAP70), among others (Fig. 5 and Table S6). The analysis revealed three main groups of intracellular proteins common to all the CLL analyzed samples and directly correlated with quantitative data, and also well‐known intracellular signaling pathways: (a) B‐cell activation pathways: as receptors of the complement system, scavenger receptors, and chemokine receptors (SCAF1, RPB1 and CXCR4, respectively); (b) BCR‐associated intracellular signaling pathways: BTK, PLCy2, LYN, SYK, and ZAP70; (c) Transcription factors related to B‐cell differentiation and proliferation: p21 (CDKN1A), ERK2 (see Fig. 5 and Table S6). Overall, the analysis confirmed that there is a common protein profile associated with 13qCLL samples.

Furthermore, 13 of these tyrosine phosphoproteins, p‐BTK (Tyr223), p‐SYK (Tyr525/526), p‐LYN (Tyr507), p‐PLCɣ2 (Tyr759), p‐AS160 (Thr 642), p‐BLNK (Tyr96), p‐SCR Family (Tyr416), p‐CD79A (Tyr182), p‐ZAP‐70 (Tyr319)/Syk (Tyr3529), p‐p38 MAPK (Thr180/Tyr182), p‐CD19 (Tyr531), p‐p44/42 MAPK (ERK 1/2) (Thr202/Tyr204), and p‐c‐CBL (Tyr700) were evaluated by WB in an independent subset of 13qCLL patient samples (*n* = 13) (Fig. 5e and Table S1).

Next, we evaluated the correlation between the phosphoproteome profiles and the mutational status of *IGHV* and the patients' CLL disease stage (using Rai and Binet classification). These clinical‐pathological parameters have prognostic value, and therefore, their correlation with certain phosphoproteins may indicate the usefulness of such proteins as biomarkers of the outcome of the disease. At first glance, nine of the targeted phosphoproteins were clearly detected in the primary stages (Binet A or B and Rai I–III classes) (Fig. 5e). Then, all the samples at the Binet B stage were positive for p‐BTK (Tyr223), p‐SYK (Tyr525/526), p‐PLCɣ2 (Tyr759), and p‐SCR‐Family (Tyr416). In samples classified as Binet stage C, the phosphoproteins p‐LYN (Tyr507), p‐AS160 (Thr 642), p‐SCR‐Family (Tyr416), and p‐CD79A (Tyr182) were not detected. A further description of these analyses is presented in Table S8.

On the contrary, the correlation between the *IGHV* mutational status and the phosphoprotein profiles was less remarkable. Notwithstanding, 80% of the patients with mutated *IGHV* showed a significant presence of p‐BTK (Tyr223); > 60% presented p‐SYK (Tyr525/526), p‐LYN (Tyr507) and p‐PLCɣ2 (Tyr759); ≥ 50% presented p‐AS160 (Thr 642), p‐CD79A (Tyr182), and p‐ZAP‐70 (Tyr319)/SYK (Tyr352); and > 30% presented p‐BLNK (Tyr96) and p‐SCR‐Family (Tyr416) (Table S8). By comparison with patients with nonmutated *IGHV*, we found that > 70% presented p‐BTK (Tyr223); > 50% presented p‐SYK (Tyr525/526), p‐PLCɣ2 (Tyr759) and p‐ZAP‐70 (Tyr319)/Syk (Tyr352); > 40% presented p‐BLNK (Tyr96) and p‐CD79A (Tyr182); and > 25% presented p‐LYN (Tyr507), p‐AS160 (Thr 642), and p‐SCR‐Family (Tyr416) (Table S8). Finally, it is worth mentioning that the phosphorylation of p‐Lyn (Tyr507) and p‐AS160 (Thr642) was significantly different between mutated and nonmutated *IGHV* groups.

### 3.4 A kinase signaling protein interaction network detected in 13qCLL, unrevealed by bioinformatics analysis

**Fig. 6:**
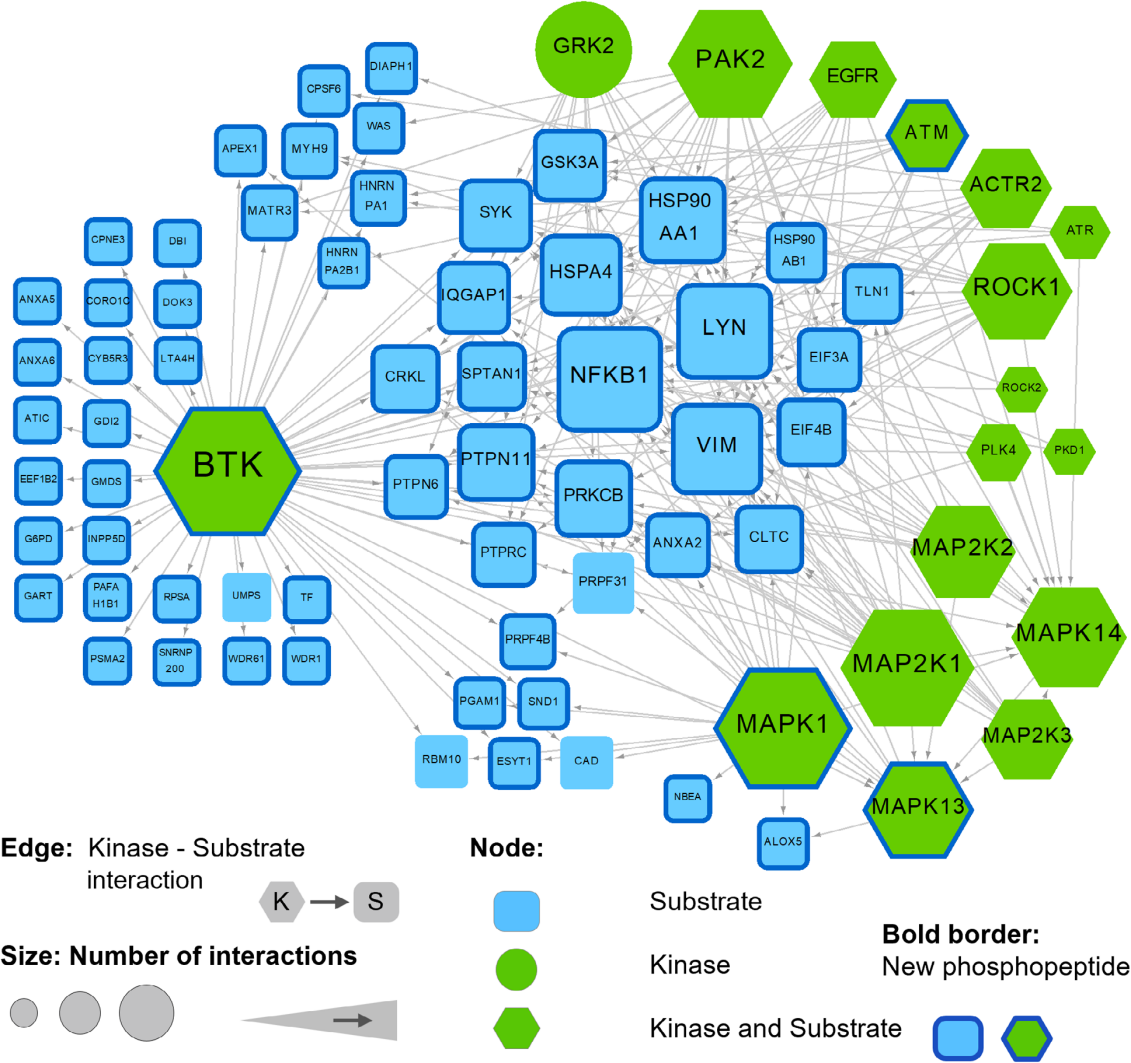
Kinase–substrate interaction network retrieved from the NetworKIN database using the proteins detected in this CLL proteomic study. The network only depicts interactions between kinases and substrates detected across all the A, B, C, and D samples in the LC–MS/MS and phosphor‐LC–MS/MS proteomic profiles of these CLL patients. Shape and node color represent the type of protein (kinase, substrate or both kinase and substrate). Node size is proportional to the number of interactions a protein is participating in. Bold border indicates the presence of phosphopeptides reported for the first time in this work.

The Kinase signaling network was retrieved from the NetworkKIN database (using database version from September 2020) [54]. NetworKIN incorporates both experimentally validated and motif‐based predicted kinase–substrate interactions [55]. From the 207 kinases collected at NetworkKIN, 20 kinases were detected in at least one CLL sample. Seventeen out of the 20 kinases (85%) were detected in the proteome of all A, B, C, and D samples. Furthermore, we can assume that the phosphoproteins detected by the LC–MS/MS experiments might be potential targets of kinase activity. From the 8121 protein kinase substrates included in NetworkKIN, 203 were detected as phosphoproteins in at least one LC–MS/MS experiment, which represents 64.2% of the total 316 phosphoproteins detected in our experiments. Moreover, 64 out of these 203 (31.5%) corresponded to phosphoproteins detected in all A–D samples. This information was used to reconstruct a CLL‐specific kinase signaling network (Fig. 6). The network represents only interactions happening between the 17 kinases (green nodes) and 60 substrates (blue nodes) detected in the proteome and phosphoproteome of all the A–D samples. Except for GRK2 kinase (circle node), all the kinases were also identified as phosphorylation substrates (hexagon shape). The network includes 77.9% phosphopeptides detected for the first time (these proteins with novel phosphopeptides are marked with a bold blue border in the network presented in Fig. 6).

## 4 Discussion

B‐CLL cells have been extensively characterized by genomic, transcriptomic, and immunophenotypic grounds. Several of these genetic alterations and phenotypic markers have proved to be of great diagnostic and/or prognostic utility [4, 5, 56, 58]. Recent studies have demonstrated the implication of multiple proteins in the development of the disease [59, 60, 61], including the phosphoproteomic profile of CLL cells [32, 33, 34, 35, 36, 37, 38].

Enhancing high‐throughput analysis of the tumor cell proteome by using high‐resolution MS/MS approaches and microarray technology might, therefore, contribute to getting a deeper insight into the disease and the potentially targetable proteins and protein networks [48, 62]. Nonetheless, such approaches should not only assess the overall (qualitative and quantitative) protein profile of tumor cells but specifically investigate the baseline functional activity of the expressed proteins, via, for example, high‐throughput evaluation of the tumor cell phosphoproteome, to get insight into those signaling pathways that might be activated and potentially targetable by existing and novel drugs [39, 63]. Despite technical developments in the field that allow usage of such assays, so far, they have mostly focused on the analysis of cell lines and/or a few specific proteins [37, 64, 65]. In our study, we employed both MS, protein array technology, and WB to leverage their complementary strengths. MS was chosen as our primary discovery platform due to its ‘hypothesis‐free’ approach, allowing for the detection of thousands of proteins simultaneously and providing a comprehensive view of the proteome [66]. Protein arrays were used as a validation technique to complement mass spectrometry (MS) data [67]. They provide higher sensitivity, targeted validation, enhanced reproducibility, precise quantification, and scalability, thereby strengthening the reliability of our findings and improving the characterization of the CLL proteome [68].

Here, we investigated the overall proteome and phosphoproteome of highly purified CLL and MBL tumor B cells directly obtained from primary patient samples. For this purpose, those peptides (and corresponding proteins) with phosphorylated tyrosine (Tyr), serine (Ser), and/or threonine (Thr) residues were purified and screened. The combination with MS‐based quantification of oncogenic B‐cell proteome enabled the characterization of signaling events potentially involved in CLL. Additionally, we used public repositories of functional ontology and kinase signaling interactions to identify key cellular pathways and reconstruct functionally meaningful associations of the CLL proteome.

Our analysis yielded a total of 2970 proteins and 316 phosphoproteins across all examined samples. While these numbers may appear modest in comparison with some published studies, it is crucial to emphasize the robustness of our identifications. We implemented a rigorous FDR threshold of < 1% at three levels: protein, peptide, and PSM, ensuring a high level of confidence in our identifications and minimizing the inclusion of false positives. Furthermore, we imposed an additional criterion that only proteins with at least two unique peptides detected were included in the final dataset. This approach substantially reduces the likelihood of spurious identifications and enhances the overall reliability of our proteomic profile. The implementation of such stringent filters inevitably results in a more conservative, yet highly dependable, set of protein and phosphoprotein identifications, providing a solid foundation for subsequent biological interpretation and hypothesis generation. Thus, data showed a consistent proteome profile between CLL and CLL‐like MBL samples. As could be expected, those proteins expressed in common in CLL/MBL tumor B cells were involved in general cell functions as well as in specific B‐cell activities. Nonetheless, multiple proteins showed expression patterns restricted to a subset of the samples. DYNL1, a protein reported to be able to neutralize the antiapoptotic activity of BCL2 and favor the expansion of the leukemic B cells, was only detected in sample A [69, 70]. Similarly, the expression of RFWD3 (a positive regulator of the stability of p53) was restricted to sample B, which corresponded to the only sample showing deletion of chromosome 13q14, including deletion of the RB1 gene, in addition to del14q32 and trisomy +12.

In sample C, 12 proteins, including PIK3R4, were found to be differentially expressed vs. all other cases. Kristensen et al. [71] previously pointed to PIK3R4 (key to initiate the autophagy process) as a potential prognostic biomarker in CLL, since they found that high expression levels of this protein were associated with more aggressive disease. In a similar trend, we found ARIH2 (also known as Triad1) differentially expressed in the CLL‐like MBL sample E. Briefly, ARIH2 acts as a tumor suppressor protein and is blocked by PTPN11, a protein that was also detected in the MBL proteome dataset but at very low expression levels (up to 20‐fold less than in the CLL samples). Interestingly, PTPN11 was also found to be phosphorylated (pPTPN11; Tyr62, Tyr546, and Tyr584 phosphosites) in all analyzed CLL samples but not in the MBL cells analyzed. Altogether, these findings suggest that in CLL samples A‐D, pPTPN11 could block the expression of ARIH2 (this protein was not detected in A–D samples) and, therefore, its suppressive leukemic effect, while the expression of ARIH2 is not blocked and remains high in MBL, blocking CLL development and progression in the later MBL (sample E) case [72].

Despite only one MBL sample being investigated, this sample was associated with the absence of expression of 293 proteins found in common in the other CLL tumor B‐cell samples. Among others, these proteins included oncogenic proteins, such as VAV1 (proto‐oncogene), BCL2 (apoptosis regulator, phosphorylated on Thr464), ZAP70 (tyrosine protein kinase used as CLL prognostic marker), CD53, CD79b (BCR‐associated protein), and CD20.

On the contrary, the phosphoproteome revealed a notably lower consistency between A and D samples when compared to the global proteome of the CLL/MBL. Because of the pivotal role of protein phosphorylation in cell signaling, this observation might shed light on the understanding of the heterogeneity of the disease, particularly in terms of genetic origin and progression. Interestingly, samples C and D (both corresponding to stage C/IV CLL cases) shared around 50% of the phosphoproteins identified, which might be considered an asset of the critical PTM in this disease.

Our analysis revealed that approximately 10% (220/2160) of proteins from the common CLL signature were phosphorylated, indicating their potential functional relevance in the disease context. These phosphorylated proteins were involved in critical cellular processes known to be dysregulated in CLL. Notably, we observed phosphorylation of key components of the tonic B‐cell receptor (BCR) signaling pathway, including BTK, LYN, PLCG2, and SYK, which are crucial for promoting cell survival and proliferation in CLL [73]. Additionally, phosphorylation of transcription factors, such as STAT1, STAT3, and NFKB1, as well as kinases like MAPK1 and PRKCB, suggests active modulation of gene expression and signaling cascades that support CLL cell survival [73]. The phosphorylation of proteins involved in apoptosis regulation further underscores the complex mechanisms contributing to the characteristic apoptosis resistance in CLL cells [74]. Interestingly, we also noted phosphorylation of proteins associated with cell adhesion and migration, such as VASP and TLN1, which may facilitate interactions with the supportive tumor microenvironment [73]. These findings collectively provide insights into the multifaceted role of protein phosphorylation in CLL pathogenesis and highlight potential targets for therapeutic intervention.

In this regard, Cochran et al. [75], O'Hayre et al. [37], and Myhrvold et al. [76] have all contributed crucial insights into the role of phosphoproteins in CLL. O'Hayre et al. [37] applied phosphoproteomics to map phosphorylation events in the CXCL12/CXCR4 signaling axis, revealing its involvement in cell migration, cytoskeletal reorganization, and interactions with the bone marrow microenvironment—key processes for CLL cell survival and trafficking. Cochran's study focused on phosphoproteins like nucleophosmin and 14‐3‐3β, highlighting their roles in regulating p53 and distinguishing mutated and unmutated CLL subtypes [75]. Myhrvold et al. [76] used single‐cell profiling to examine phosphoprotein levels, providing insights into the molecular heterogeneity of CLL cells, with implications for patient stratification and targeted therapies.

In our study, functional enrichment analysis of 2160 proteins commonly expressed in 13qCLL/MBL tumor cells identified critical phosphoproteins involved in BCR signaling, immune response regulation, and cell cycle regulation, such as BTK, PRKCB, STAT1, and SYK. Additionally, a heatmap analysis uncovered significant alterations in leukocyte function, neutrophil dysregulation, and proteasome activity. Alterations in B‐cell‐associated proteins like SYK, BLNK, and ATM were observed, alongside changes in RNA splicing pathways. Fifty‐five out of the 594 phosphopeptides identified in this study had never been reported before, marking a significant contribution to the existing database of phosphoproteins in CLL, further expanding the knowledge on CLL pathogenesis. These findings underscore the importance of phosphoproteomics in uncovering novel targets for CLL diagnosis and therapy.

Notably, among the kinases phosphorylated across all samples, we found proteins associated with various signal transduction pathways involved in the pathogenesis of CLL, including BCR signaling, chemokine receptor signaling, and toll‐like receptor (TLR) signaling pathways [77, 78, 79, 80, 81]. Of note, in all the studied CLL cells, the chemokine receptor and TLR signaling pathways were underrepresented (phosphorylation being restricted in common to the CXCR4, STAT3, and TLR1 proteins) vs. the BCR signaling pathway. Thus, many BCR‐related proteins were found to be phosphorylated in all or the majority of samples (LYN, SYK, PI3K, BTK, ZAP70, PLCG3, ERK, NFAT, CD19, PRKCB, NF‐Kβ, JNK, and VAV) [82, 83, 84, 85, 86, 87], suggesting that BCR signaling plays a critical role in the maintenance/survival of CLL/MBL tumor cells (Fig. 5). In line with previous observations [87], several proteins involved in the NF‐βB and STAT3 signaling pathway related to CLL cell activation, proliferation, survival, adhesion, and homing were also found to be increased in common in CLL samples (samples A–D) at significantly higher levels than in CLL‐like MBL cells (i.e., sample E), suggesting a potential role for this specific signaling pathway in the malignant transformation of the disease.

Comparing the results from the global phosphoproteome analysis and the affinity proteomics, several similarities and differences emerged that highlight the strengths and limitations of each approach. Both analyses identified key proteins involved in BCR signaling, such as BTK, LYN, SYK, and ZAP70, underscoring the critical role of these proteins in CLL pathogenesis. Additionally, both methods revealed heterogeneity in phosphoprotein profiles across 13qCLL samples, indicating variability in phosphorylation patterns that may influence disease progression and response to therapy. However, the global phosphoproteome analysis identified a total of 594 phosphopeptides corresponding to 316 phosphoproteins, while the affinity proteomics platform focused on a more targeted set of proteins. Furthermore, the global analysis revealed two distinct groups of samples based on their phosphoproteomic signatures, suggesting a more nuanced understanding of sample heterogeneity. This distinction emphasizes how each method can complement one another; while the global analysis provides a comprehensive view of phosphorylation across multiple residues, the use of protein microarrays focuses on critical preselected aspects of signaling pathways to targeted validate them. Together, these approaches can enhance our understanding of CLL biology and may inform future therapeutic strategies.

Finally, our results highlighted the significant involvement of the BCR and NF‐kβ/STAT3 pathways, suggesting promising opportunities for drug repurposing in treating CLL. One critical target is BTK, a key player in tonic BCR signaling. Established BTK inhibitors, such as ibrutinib and acalabrutinib, have already shown efficacy in treating CLL. Inhibitors that target SYK, such as fostamatinib, may be considered for repurposing in CLL treatment, particularly for patients who exhibit resistance to BTK inhibitors. Moreover, inhibitors targeting the NF‐kβ and STAT3 pathways, which are implicated in various malignancies, could also be repurposed for CLL. For instance, bortezomib and ruxolitinib (a JAK1/2 inhibitor targeting STAT3) have shown promise in other hematologic cancers and may enhance therapeutic strategies when combined with BCR inhibitors. Also, inhibitors of GSK3, such as lithium or newer selective GSK3 inhibitors, could be explored for their potential effects on CLL cell survival. Beyond these pathways, HSP90AA1 and HSP90AB1 are critical chaperone proteins that stabilize several oncogenic proteins. Inhibitors like geldanamycin or ganetespib could be repurposed to target these proteins, potentially disrupting the stability of multiple signaling pathways involved in CLL. Overall, the integration of these therapies could lead to more effective treatment regimens by simultaneously targeting multiple pathways involved in disease progression. In addition to exploring existing drugs, the study's identification of 55 novel phosphopeptides presents an opportunity to investigate new therapeutic targets. Further research into these phosphoproteins may uncover additional candidates for targeted therapies that specifically address the unique signaling landscape of CLL. By leveraging the insights gained from this phosphoproteomic analysis, future investigations might develop innovative treatment strategies that not only improve patient outcomes but also minimize resistance mechanisms commonly associated with current therapies.

## 5 Conclusions

In summary, these results provided an integrated MS‐based map of the CLL global proteome and phosphoproteome and showed that CLL/MBL tumor B cells from different patients display a high degree of overlap in protein expression but highly diverse phosphoproteome profiles. Despite the limited sample size, our comprehensive proteomic and phosphoproteomic analyses revealed consistent protein expression patterns and identified novel phosphopeptides across samples. We successfully identified 55 novel phosphopeptides, expanding our understanding of the phosphoproteome in this context. Furthermore, we employed a multifaceted approach by integrating diverse proteomics‐based techniques, including LC–MS/MS, protein microarrays, and western blotting. This comprehensive strategy allowed for a thorough characterization of B cells from CLL patients, providing a more holistic view of the cellular proteome and its modifications in this disease state. The functional analysis reinforced the pivotal role of tonic BCR signaling pathway in CLL profile. The functional characterization and the kinase network reconstruction provided novel insights into pathological B‐cell signaling and highlighted multiple proteins as potential biomarkers of disease progression. Further studies in larger patient series and normal B‐cell populations are needed to confirm these findings. Nonetheless, this work represents a first step to integrate multifaceted high‐throughput analyses to simultaneously characterize the proteome and phosphoproteome of CLL.

## Conflict of interest

The authors declare no conflict of interest.

## Author contributions

MF and JDLR contributed to the conceptualization. PD, PJ‐V, AL‐V, MA, AN‐B, JMB, and MB contributed to the methodology. CD, MLG‐V, HF‐G, SM‐H, JMS‐S, JDLR, MG‐D, JA, and AO contributed to the formal analysis. PD, PJ‐V, AL‐V, MA, and AN‐B contributed to the investigation. MA, AN‐B, JM‐B, MB, MG‐D, JA, AO, and MF contributed to the resources. PD, P‐JV, and MF contributed to the writing—original draft preparation. PD, PJ‐V, AL‐V, MA, AN‐B, MGV, MG, RG, and MF contributed to the writing—review and editing. CD, MG‐V, HF‐G, SM‐H, JMS‐S, and JDLR contributed to the visualization. MF and RG contributed to the supervision. MF contributed to the project administration and funding acquisition. All authors have read and agreed to the published version of the manuscript.

## Supporting information


**Fig. S1.** SDS/PAGE gels. (A) Coomassie staining. (B) Ponceau S staining. (C) Western blot for pMAPK/CDK detection. (D) Western blot for pTyr detection.
**Fig. S2.** Affinity Proteomics images. (A) Image from Iris™ Optical Quality Control JetSpider (after printing and before sample incubation). (B) genepix®pro 6.0 software array image with a description of possible results.
**Fig. S3.** (A) Rocket plot of relative expression of 18 082 genes in healthy and CLL samples. Dot color indicates the number of LC–MS/MS experiments in which the protein was also quantified. (B) Density plots of microarray signal profile distinguishing the genes also identified at LC–MS/MS experiments.
**Fig. S4.** Degree of correlation and similarity both for intra‐sample and inter‐sample comparisons. (A). CLL/MBL samples A, B, C, D and E for proteome characterization. (B). CLL/MBL samples A, B, C, D and E for phosphoproteome characterization.

**Table S1.** Table of clinical‐biological characteristics from 19 (18 CLL patients and 1 CLL‐like MBL) patients.

**Table S2.** Antibodies list used in Protein Microarrays and Immunoblotting.

**Table S3.** Proteome characterization results of LC–MS/MS.

**Table S4.** Functional pathway analysis for the proteins expressed in CLL/MBL tumor cells.

**Table S5.** Proteome and phosphoproteome heatmaps and clusters results of LC–MS/MS.

**Table S6.** Proteome and phosphoproteome heatmaps and clusters results of Protein Microarrays.

**Table S7.** Phosphoproteome characterization results of LC–MS/MS.

**Table S8.** Table summarizing the western blot results from nine phosphoproteins involved in BCR signaling in 13 CLL cell lysate samples as well as crosstabs from Binet and Rai Stage and *IGHV* mutational status results between samples.

## Data Availability

The mass spectrometry proteomics data have been deposited to the ProteomeXchange Consortium via the PRIDE partner repository with the dataset identifier PXD005997 [88].
