# Supplementary material for: Tonic signaling of the B‐cell antigen‐specific receptor is a common functional hallmark in chronic lymphocytic leukemia cell phosphoproteomes at early disease stages
Source: Mol Oncol. 2025 Mar 25;19(12):3706–28. doi: 10.1002/1878-0261.70032 (PMC12688175; doi:10.1002/1878-0261.70032)
Supplement: Supplementary file 1 — Fig. S1. SDS/PAGE gels. (A) Coomassie staining. (B) Ponceau S staining. (C) Western blot for pMAPK/CDK detection. (D) Western blot for pTyr detection. Fig. S2. Affinity Proteomics images. (A) Image from Iris™ Optical Quality Control JetSpider (after printing and before sample incubation). (B) genepix®pro 6.0 software array image with a description of possible results. Fig. S3. (A) Rocket plot of relative expression of 18 082 genes in healthy and CLL samples. Dot color indicates the number of LC–MS/MS experiments in which the protein was also quantified. (B) Density plots of microarray signal profile distinguishing the genes also identified at LC–MS/MS experiments. Fig. S4. Degree of correlation and similarity both for intra‐sample and inter‐sample comparisons. (A). CLL/MBL samples A, B, C, D and E for proteome characterization. (B). CLL/MBL samples A, B, C, D and E for phosphoproteome characterization. [file MOL2-19-3706-s003.docx]

# Supplementary Figures and Tables

# Supplementary Figures


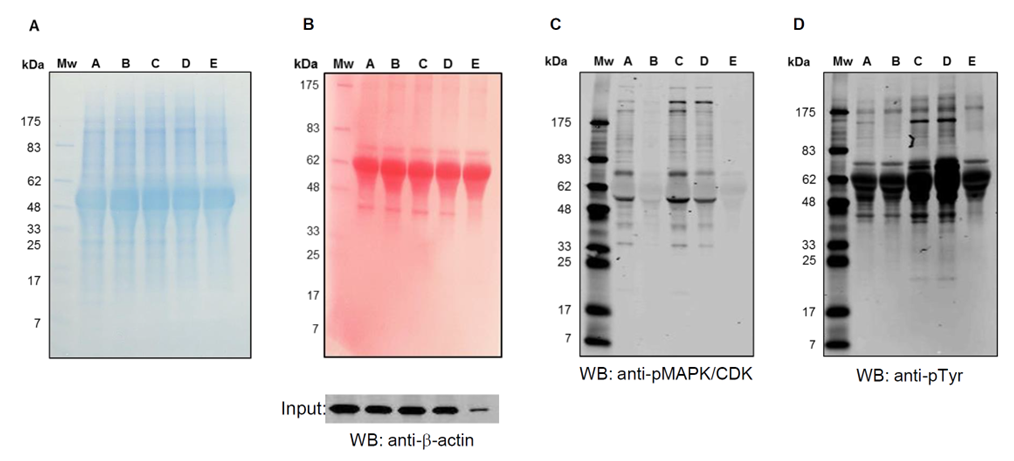


**Supplementary Figure S1.** SDS-PAGE gels. (A) Coommasie staining. (B) Ponceau S staining. (C) Western blot for pMAPK/CDK detection. (D) Western blot for pTyr detection.


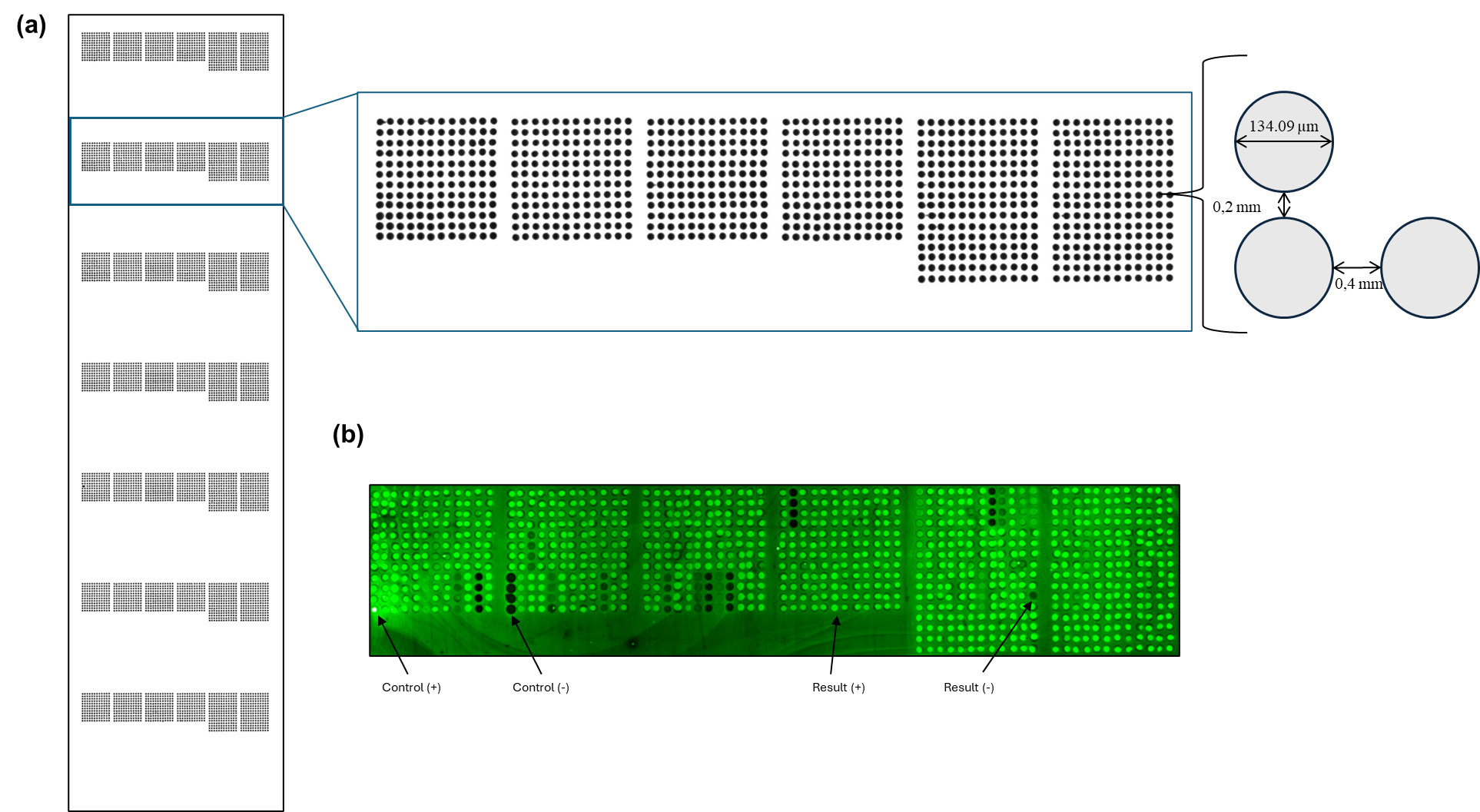


**Supplementary Figure S2.** Affinity Proteomics images. A. Image from Iris ™ Optical Quality Control JetSpider (after printing and before sample incubation). B. GenePix®Pro 6.0 software array image with a description of possible results.


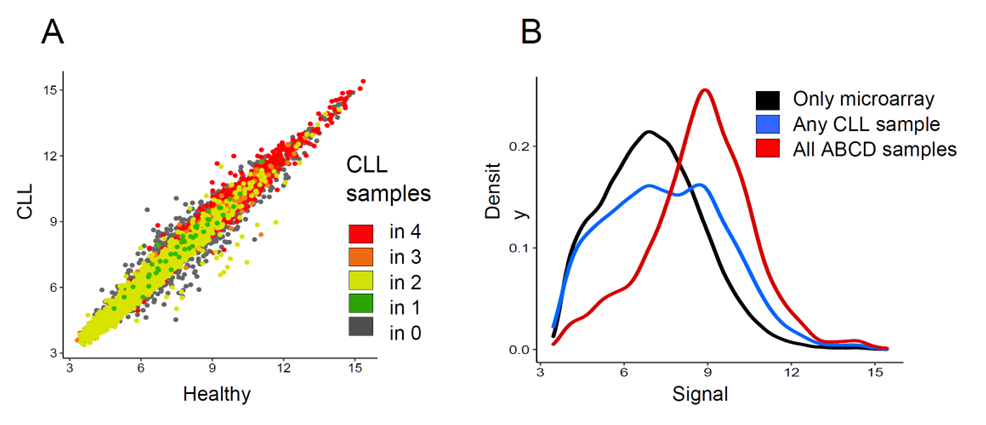


**Supplementary Figure S3.** (A) Rocket plot of relative expression of 18,082 genes in healthy and CLL samples. Dot color indicates the number of LC-MS/MS experiments in which the protein was also quantified. (B) Density plots of microarray signal profile distinguishing the genes also identified at LC-MS/MS experiments.


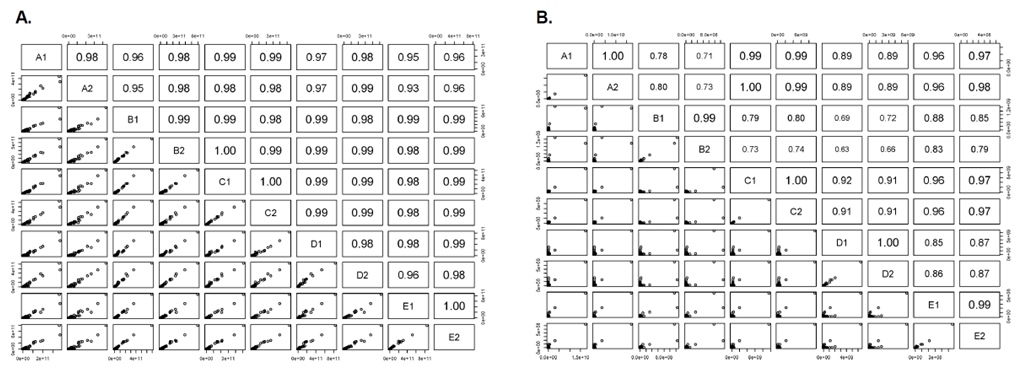


**Supplementary Figure S4.** Degree of correlation and similarity both for intra-sample and inter-sample comparisons. (A). CLL/MBL samples A, B, C, D and E for proteome characterization. (B). CLL/MBL samples A, B, C, D and E for phosphoproteome characterization.

## Supplementary Tables

**Supplementary Table S1.** Table of clinical-biological characteristics from 19 (18 CLL patients and 1 CLL-like MBL) patients.

**Supplementary Table S2.** Antibodies list used in Protein Microarrays and Immunoblotting.

**Supplementary Table S3.** Proteome characterization results of LC-MS/MS.

**Supplementary Table S4.** Functional pathway analysis for the proteins expressed in CLL/MBL tumor cells.

**Supplementary Table S5.** Proteome and phosphoproteome heatmaps and clusters results of LC-MS/MS.

**Supplementary Table S6.** Proteome and phosphoproteome heatmaps and clusters results of Protein Microarrays.

**Supplementary Table S7.** Phosphoproteome characterization results of LC-MS/MS.

**Supplementary Table S8.** Table summarizing the western blot results from 9 phosphoproteins involved in BCR signaling in 13 CLL cell lysate samples as well as crosstabs from Binet and Rai Stage and IGHV mutational status results between samples.
